# The efficacy and safety profile of 2-weekly dosing of bevacizumab-containing chemotherapy for platinum-resistant recurrent ovarian cancer

**DOI:** 10.1007/s10147-021-01996-8

**Published:** 2021-07-26

**Authors:** Masayuki Sekine, Takayuki Enomoto, Yoh Watanabe, Hidetaka Katabuchi, Nobuo Yaegashi, Daisuke Aoki

**Affiliations:** 1 Department of Obstetrics and Gynecology, Niigata University Graduate School of Medical and Dental Sciences, 757 Ichibancho, Asahimachi-dori, Chuo Ward, Niigata, Niigata Japan; 2 Division of Obstetrics and Gynecology, Faculty of Medicine, Tohoku Medical and Pharmaceutical University, 4-4-1 Komatsushima, Aobaku, Sendai, Miyagi Japan; 3 Department of Obstetrics and Gynecology, Faculty of Life Sciences, Kumamoto University, 1-1-1 Honjo, Chuo-ku, Kumamoto, Kumamoto Japan; 4 Department of Obstetrics and Gynecology, Tohoku University Graduate School of Medicine, 2-1 Seiryo-machi, Aoba-ku, Sendai, Miyagi Japan; 5 Department of Obstetrics and Gynecology, Keio University School of Medicine, 35 Shinanomachi, Shinjuku-ku, Tokyo, Japan

**Keywords:** Bevacizumab, Drug-related side effects and adverse reactions, Drug therapy, Ovarian neoplasms

## Abstract

**Background:**

Despite being widely used, to date (June 2021), the regimen of bevacizumab 10 mg/kg every 2 weeks (Q2W) combined with chemotherapy is not approved in Japan for patients with platinum-resistant recurrent ovarian cancer. In this retrospective analysis, we evaluated the usage patterns of bevacizumab administered for platinum-resistant recurrent ovarian cancer.

**Methods:**

We obtained clinical data from 155 Japanese medical facilities between November 2013 and December 2018 via a survey. Items included the number of cases of platinum-resistant recurrent ovarian cancer treated with bevacizumab according to dosage. For regimens including bevacizumab 10 mg/kg Q2W, additional information was requested relating to concomitantly administered agents, and the efficacy and safety of the regimen.

**Results:**

Of 1739 bevacizumab-containing regimens reported in 1633 patients with recurrent ovarian cancer, 264 used 10 mg/kg Q2W. The overall response rate (ORR) with this regimen was 26.1%. Response rates varied according to regimen and were particularly favorable when bevacizumab 10 mg/kg Q2W was administered with paclitaxel (ORR, 53.0%) versus liposomal doxorubicin (15.0%; *P* < 0.0001) and irinotecan (7.7%; *P* < 0.028). The most frequent Grade ≥ 3 adverse events associated with bevacizumab 10 mg/kg Q2W were neutropenia (11.7%) and hypertension (11.7%). The most frequent bevacizumab-associated Grade ≥ 3 adverse events with bevacizumab plus paclitaxel versus bevacizumab plus liposomal doxorubicin were hypertension (9.0% versus 13.9%) and proteinuria (3.0% versus 8.4%).

**Conclusions:**

Bevacizumab 10 mg/kg Q2W appears efficacious for patients with recurrent ovarian cancer, with a manageable toxicity profile. Approval of this regimen is clinically desirable for Japanese patients with ovarian cancer.

## Introduction

According to the World Health Organization, in 2020 there were 10,964 new cases of ovarian cancer diagnosed in Japan and 5302 deaths from the disease [1]. The incidence of ovarian cancer is increasing [2, 3], and its mortality exceeds that of any other type of gynecologic cancer [1, 2]. Ovarian cancer is usually treated with surgery combined with chemotherapy [2], but many patients develop recurrence due to residual micrometastases even after macroscopic complete remission is achieved [4, 5]. Recurrence occurs in 55% of patients with late-stage ovarian cancer (stage III and stage IV) within 2 years and in 70% within 5 years [6].

For patients who develop recurrence within 6 months of their final treatment, platinum-based chemotherapy is not recommended [7]. For patients with platinum-resistant disease, bevacizumab, a monoclonal antibody targeting vascular endothelial growth factor, has demonstrated antitumor activity [7, 8]. Bevacizumab has been approved in several countries to be used in combination with chemotherapy, followed by maintenance monotherapy [9–11].

The efficacy and safety of bevacizumab (at doses of 15 mg/kg every 3 weeks [Q3W] or 10 mg/kg every 2 weeks [Q2W]) combined with chemotherapy were demonstrated in patients with platinum-resistant recurrent ovarian cancer in the phase III AURELIA trial conducted in Europe [12]. Although both of these dosing regimens have been approved in Europe and North America [9, 10], only the 15 mg/kg dose Q3W or longer has been approved in Japan [11]. Nonetheless, despite the lack of official approval, bevacizumab 10 mg/kg Q2W is used in actual clinical practice in Japan, because the chemotherapy regimen for platinum-resistant recurrent ovarian cancer is typically every 2 or 4 weeks; thus, the Q2W administration of bevacizumab is easy to implement for oncologists and their patients. Moreover, bevacizumab 10 mg/kg Q2W in combination with carboplatin and pegylated liposomal doxorubicin was shown to be efficacious in a group of patients with platinum-sensitive recurrent ovarian cancer in the recently published AGO-OVAR 2.21 trial [13]. As a result, the Q2W regimen is widely cited in textbooks [14, 15] and guidelines [7, 16, 17] both in Japan and overseas.

We conducted a real-world analysis of Japanese clinical practice to reveal the usage patterns of bevacizumab for platinum-resistant recurrent ovarian cancer, and to examine the efficacy and safety of bevacizumab 10 mg/kg Q2W.

## Materials and methods

### Study design

This was a retrospective analysis of clinical practices within Japan; no patient identifying data were collected, and no interventions or procedures were mandated. This study was approved by the Ethics Committee of the primary study facility (Niigata University, Niigata, Japan) and the Ethics Committees of all other participating facilities.

A questionnaire was sent by postal mail to 388 medical facilities across Japan between October 2018 and December 2018. Information was requested on the usage patterns of bevacizumab administered for platinum-resistant recurrent ovarian cancer between November 2013 and December 2018. The selection of facilities to which questionnaires were sent was based on their identification as a workplace for specialists certified by the Japan Society of Gynecologic Oncology, and where bevacizumab was likely to be offered as a treatment for patients with ovarian cancer.

### Survey items

The items in the questionnaire included the total number of cases of platinum-resistant recurrent ovarian cancer treated with bevacizumab and the number of cases according to bevacizumab dosage. For regimens including bevacizumab 10 mg/kg Q2W, additional information was requested on the types and dosages of concomitantly administered anti-cancer agents, the efficacy of the treatment regimen (based on the Response Evaluation Criteria in Solid Tumors [RECIST] version 1.1), and the safety of the regimen (based on adverse events [AEs]).

### Data analysis

As this analysis was not designed to statistically test a hypothesis, no power calculations were performed. All data collected were summarized descriptively. We used R version 3.4.1 (Vienna, Austria) to calculate Fisher’s exact test, and a two-sided *P* value of < 0.05 was considered to indicate statistical significance.

## Results

### Bevacizumab regimens used in real-world clinical practice

Of the 388 facilities who were sent the questionnaire, 155 provided a response (39.9% response rate). Treatment details were received for 1633 patients with recurrent ovarian cancer; the total number of bevacizumab regimens administered was 1739 (Table 1). The most common regimen was 15 mg/kg Q3W (997 regimens), followed by 15 mg/kg every 4 weeks (476 regimens). Of the 155 facilities which responded, 41 (26.5%) were using the dosage of interest (10 mg/kg Q2W); overall, this dosage was used in 264 regimens in 264 patients.

**Table 1:** Regimens of bevacizumab used in real-world clinical practice to treat platinum-resistant recurrent ovarian cancer

Regimen, *n*	Agents combined with bevacizumab					Overall number of regimens^b^
	Liposomal doxorubicin	Paclitaxel	Gemcitabine	Topotecan	Other^a^	
Total	602	613	284	182	214	1739
15 mg/kg every 3 weeks	209	429	233	130	127	997
15 mg/kg every 4 weeks	217	111	44	52	61	476
10 mg/kg every 2 weeks	176	73	7	0	24^c^	264
Other^d^	0	0	0	0	2	2

^a^Including bevacizumab monotherapy

^b^When several regimens were used for a single patient, those regimens were counted individually

^c^Irinotecan (*n* = 16), cisplatin (*n* = 7), and carboplatin (*n* = 1)

^d^15 mg/kg administered on a flexible schedule every 3 or 4 weeks according to the condition of the patient

As shown in Table 1, the agents most commonly administered alongside bevacizumab (any regimen) were paclitaxel (613 regimens) and liposomal doxorubicin (602 regimens). Bevacizumab 10 mg/kg Q2W was administered most frequently with liposomal doxorubicin (176 regimens), paclitaxel (73 regimens), irinotecan (16 regimens), or gemcitabine (7 regimens). The most common regimens for each concomitant agent were as follows: liposomal doxorubicin 40 mg/m^2^, administered on day 1 of a 4-week cycle (*n/N* = 166/176); paclitaxel 80 mg/m^2^, administered on day 1 of a 1-week cycle (*n/N* = 68/73); irinotecan 70 mg/m^2^, administered on days 1, 8, and 15 of a 4-week cycle (*n/N* = 7/16); and gemcitabine 1000 mg/m^2^, administered on days 1 of a 2-week cycle (*n/N* = 5/7).

### Response rates with bevacizumab 10 mg/kg Q2W

Table 2 shows the best overall response achieved with bevacizumab 10 mg/kg Q2W for platinum-resistant recurrent ovarian cancer, based on RECIST v1.1 criteria; the overall response rate (ORR) with this regimen was 26.1%. Rates varied according to the combination regimen used and were particularly favorable when bevacizumab 10 mg/kg Q2W was combined with paclitaxel (ORR, 53.0%) versus liposomal doxorubicin (15.0%; *P* < 0.0001) and irinotecan (7.7%; *P* < 0.028).

**Table 2:** Efficacy of bevacizumab 10 mg/kg every 2 weeks for platinum-resistant recurrent ovarian cancer (based on the Response Evaluation Criteria in Solid Tumors version 1.1)

Best overall response, *n* (%)	Agents combined with bevacizumab^a^			All bevacizumab 10 mg/kg (*n* = 264*)*
	Liposomal doxorubicin (*n* = 167)	Paclitaxel (*n* = 66)	Irinotecan (*n* = 13)	
Complete response	3 (1.8)	1 (1.5)	0	4 (1.5)
Partial response	22 (13.2)	34 (51.5)	1 (7.7)	65 (24.6)
Overall response rate^b^	25 (15.0)	35 (53.0)	1 (7.7)	69 (26.1)
Stable disease	49 (29.3)	15 (22.7)	1 (7.7)	69 (26.1)
Disease control rate^c^	74 (44.3)	50 (75.8)	2 (15.4)	138 (52.3)
Progressive disease	74 (44.3)	13 (19.7)	7 (53.8)	99 (37.5)
Not assessed/unknown	19 (11.4)	3 (4.5)	4 (30.8)	27 (10.2)

^a^Data for the best overall response achieved without switching agents is presented

^b^Complete response + partial response

^c^Complete response + partial response + stable disease

Overall, there were 4 complete responses (bevacizumab plus liposomal doxorubicin, *n* = 3; bevacizumab plus paclitaxel, *n* = 1) and 65 partial responses (including 22 with bevacizumab plus liposomal doxorubicin and 34 with bevacizumab plus paclitaxel). The disease control rate (complete response + partial response + stable disease) was 52.3% with bevacizumab 10 mg/kg Q2W; this varied from 75.8% when bevacizumab was combined with paclitaxel to 15.4% when combined with irinotecan.

### Safety profile of bevacizumab 10 mg/kg Q2W

AEs of Grade ≥ 3 occurring in ≥ 1% of patients receiving bevacizumab 10 mg/kg Q2W are shown in Table 3. Overall, 154 AEs of Grade ≥ 3 were reported with bevacizumab 10 mg/kg Q2W. The most frequent were neutropenia (*n* = 31 [11.7%]), hypertension (*n* = 31 [11.7%]), proteinuria (*n* = 16 [6.1%]), and anemia (*n* = 14 [5.3%]). Most (*n* = 134) were Grade 3; only a single Grade 5 event was reported, which was acute respiratory distress syndrome.

**Table 3:** Adverse events of Grade ≥ 3 occurring in ≥ 1% of patients receiving bevacizumab 10 mg/kg every 2 weeks (*n* = 264)

Adverse event, *n* (%)	All events of Grade ≥ 3^a^	Grade 3	Grade 4
Any adverse event	154 (58.3)	134 (50.8)	19 (7.2)
Hematologic toxicities			
Neutropenia	31 (11.7)	23 (8.7)	8 (3.0)
Anemia	14 (5.3)	14 (5.3)	0
Thrombocytopenia	7 (2.7)	6 (2.3)	1 (0.4)
Febrile neutropenia	3 (1.1)	3 (1.1)	0
Non-hematologic toxicities			
Hypertension	31 (11.7)	31 (11.7)	0
Proteinuria	16 (6.1)	16 (6.1)	0
Hand–foot syndrome	6 (2.3)	6 (2.3)	0
Ileus	5 (1.9)	4 (1.5)	1 (0.4)
Gastrointestinal perforation	4 (1.6)	0	4 (1.2)
Venous thrombosis^b^	4 (1.6)	4 (1.6)	0
Mucositis	3 (1.1)	3 (1.1)	0

Adverse events were categorized according to the Preferred Terms of the Medical Dictionary for Regulatory Activities version 21.1, and graded according to the Common Terminology Criteria for Adverse Events version 4.0

^a^Only one Grade 5 event (0.4%) was reported: acute respiratory distress syndrome

^b^Includes pulmonary embolism

The safety profile (Grade ≥ 3 AEs) with bevacizumab 10 mg/kg Q2W according to combination regimen (bevacizumab plus liposomal doxorubicin versus bevacizumab plus paclitaxel) is shown in Table 4. For bevacizumab plus liposomal doxorubicin versus bevacizumab plus paclitaxel, 118 versus 29 AEs of Grade ≥ 3 were reported, and the most frequent bevacizumab-associated Grade ≥ 3 AEs were hypertension (*n* = 24 [13.9%] versus *n* = 6 [9.0%], respectively) and proteinuria (*n* = 14 [8.4%] versus *n* = 2 [3.0%], respectively).

**Table 4:** Adverse events of Grade ≥ 3 occurring with bevacizumab 10 mg/kg every 2 weeks according to combination regimen (bevacizumab plus liposomal doxorubicin versus bevacizumab plus paclitaxel)

Adverse event, *n* (%)	Events of Grade ≥ 3 occurring with bevacizumab		
	With liposomal doxorubicin (*n* = 167)	With paclitaxel (*n* = 66)	All (*n* = 264*)*
Any adverse event	118 (70.7)	29 (43.9)	154 (58.3)
Hypertension	24 (13.9)	6 (9.0)	31 (11.7)
Proteinuria	14 (8.4)	2 (3.0)	16 (6.1)
Gastrointestinal perforation	2 (1.2)	2 (3.0)	4 (1.6)
Venous thrombosis^a^	2 (1.2)	2 (3.0)	4 (1.6)

Adverse events were categorized according to the Preferred Terms of the Medical Dictionary for Regulatory Activities version 21.1 and graded according to the Common Terminology Criteria for Adverse Events version 4.0

^a^Includes pulmonary embolism

## Discussion

Although the regimen of bevacizumab 10 mg/kg Q2W is not yet (as of June 2021) approved for treatment of platinum-resistant ovarian cancer in Japan, our data indicate that such a regimen is used in around 15% (264/1739) of bevacizumab treatment regimens in this patient population. The chemotherapeutic agents most frequently combined with bevacizumab 10 mg/kg Q2W were liposomal doxorubicin and paclitaxel; this is consistent with the agents used in the phase III AURELIA trial [12]. However, unlike the AURELIA trial, no patients in our analysis were found to have received bevacizumab 10 mg/kg Q2W plus topotecan. This is likely because topotecan has dose-limiting myelosuppressive effects [18], and Japanese clinicians prefer to use an alternative topoisomerase I inhibitor, irinotecan.

Our investigation of the efficacy of bevacizumab 10 mg/kg Q2W in ovarian cancer found that the ORR for this regimen was 26.1%. This was in line with the data reported from the AURELIA trial, where the ORR with bevacizumab 10 mg/kg Q2W plus chemotherapy was 27.3% [12], and with the data from a phase II study of bevacizumab 10 mg/kg Q2W plus topotecan, where the ORR was 25.0% [19]. The present study also indicated wide differences in the ORR depending on the combination chemotherapy agent used (53.0% with paclitaxel, 15.0% with liposomal doxorubicin, 7.7% with irinotecan) with a significantly higher response rate for the paclitaxel combination compared with liposomal doxorubicin (*P* < 0.0001) and irinotecan (*P* < 0.028). Similar differences in efficacy according to the chemotherapeutic agent administered alongside bevacizumab 10 mg/kg Q2W were reported in a recent study in Korea [20], indicating that the choice of combination chemotherapy must be a key consideration in clinical practice. In the AURELIA trial, of the chemotherapeutic agents used in combination with bevacizumab 10 mg/kg Q2W, only liposomal doxorubicin was used at the dosage approved in Japan [12]; thus, further investigation of the optimal combination regimen in Japanese patients is warranted.

The occurrence of Grade ≥ 3 AEs in phase III clinical trials of bevacizumab in advanced ovarian cancer was evaluated in a previously published systematic review [21], with hypertension, thromboembolic events, proteinuria, bleeding and gastrointestinal events found to occur at a higher incidence with bevacizumab than in the control groups. Similar data were reported in Japan for the dosages of 15 mg/kg Q3W (approved for ovarian cancer) and 10 mg/kg Q2W (approved for colorectal cancer, non-small cell lung cancer, breast cancer, malignant glioma, and cervical cancer) [11]. An updated evaluation, including data from the AURELIA [12] and GOG-0218 [22] studies, plus data from the recent JGOG3022 study in Japanese patients [23], is shown in Table 5. Notably, lower rates of hypertension and higher rates of proteinuria were observed in the Japanese patients in the JGOG3022 study compared with the Western patients in the phase III trials; a similar tendency was observed in the current analysis. Overall, however, the safety profile of bevacizumab 10 mg/kg Q2W for ovarian cancer found in the present study was consistent with previous reports, with generally low rates of gastrointestinal perforation, venous thrombosis, fistula, and bleeding, and the majority of the toxicities reported can be adequately managed by gynecologic oncologists [24].

**Table 5:** Summary of selected adverse events occurring with bevacizumab in the present analysis compared with previously published studies

	Current analysis^a^	AURELIA [12]^a^	GOG-0218 [22]^b^		JGOG3022 [23]^a^	
Bevacizumab dose	10 mg/kg Q2W	15 mg/kg Q3W	15 mg/kg Q3W		15 mg/kg Q3W	
Analysis group	All patients (*n* = 264)	Bevacizumab plus chemotherapy^c^ (*n* = 179)	Bevacizumab plus paclitaxel and carboplatin (*n* = 607)	Maintenance bevacizumab (*n* = 608)	Bevacizumab plus paclitaxel and carboplatin (*n* = 293)	Maintenance bevacizumab (*n* = 293)
Adverse event, %						
Hypertension	11.7	7.3	16.5	22.9	14.0	9.2
Proteinuria	6.1	1.7	0.7	1.6	2.7	9.9
GI events	–	–	2.8	2.6	–	
Perforation	1.6	1.7	–	–	0	0.3
Fistula	0	1.1	–	–	0.3	0.3
Venous thrombosis	1.6	2.8	5.3	6.7	0.7	0.7
Bleeding	0	1.1	1.3	2.1	0	0

GI gastrointestinal; Q2W every 2 weeks; Q3W every 3 weeks

^a^Includes adverse events of Grade ≥ 3 only

^b^Events of Grade ≥ 2 were reported for hypertension; events of Grade ≥ 3 for proteinuria and bleeding; and all grade events for other items

^c^Either pegylated liposomal doxorubicin, weekly paclitaxel, or topotecan

When the rate of bevacizumab-associated AEs was compared between different chemotherapeutic combinations, a slightly higher rate of Grade ≥ 3 hypertension was found to be associated with bevacizumab plus liposomal doxorubicin (13.9%) compared with bevacizumab plus paclitaxel (9.0%); however, incidences of proteinuria, deep vein thrombosis, and gastrointestinal perforation were similar between regimens. Therefore, depending on the concomitant agents administered with bevacizumab, physicians should take appropriate precautions and implement suitable monitoring. For patients who receive a bevacizumab 10 mg/kg Q2W regimen, paclitaxel is most likely to be administered once weekly and AEs can be identified early in the course of treatment; this early identification and amelioration likely contributes to the small numbers of Grade ≥ 3 AEs reported in this analysis. Based on the reported safety profiles for liposomal doxorubicin [25] and paclitaxel [26] in Japanese patients, hypertension (all grades) would be expected to occur in 5.4% and 12.7% of treated patients, respectively, and Grade ≥ 3 hypertension in 0% and 1.1%, respectively. Notably, hypertension occurs more frequently in Japanese patients receiving weekly paclitaxel. There are no clear differences in the frequency of proteinuria expected with liposomal doxorubicin and paclitaxel in Japanese patients, with all grade events observed in 13.5% and 12.7%, respectively, and Grade ≥ 3 proteinuria in 0% and 0.6%, respectively.

Together, the results of our study showed that Japanese oncologists use a variety of chemotherapeutic agents for platinum-resistant ovarian cancer combined with bevacizumab 10 mg/kg Q2W, and the efficacy and safety of each combination vary considerably. Overall, however, the data indicate that bevacizumab 10 mg/kg Q2W can be efficacious for patients with recurrent ovarian cancer and has a well-understood toxicity profile which can be managed by clinicians. The approval of this dosage regimen in Japan is keenly awaited, as it is well suited for clinical practice, and may reduce the treatment burden on patients by streamlining hospital visits. Once approved, bevacizumab 10 mg/kg Q2W will offer patients with platinum-resistant ovarian cancer more treatment choices, and in the future it may be used in new combination therapies with approved or developmental agents in Japan.

The study has some limitations which must be considered. First, this was a questionnaire survey which included selected medical facilities. Although the institutions invited to participate were those expected to have a high volume of bevacizumab-treated patients, we cannot be certain that the data reported are representative of all ovarian cancer treatment across Japan. Second, it is possible that our data are biased in favor of sites who used bevacizumab 10 mg/kg Q2W. The rate of collected questionnaires was not as high as we would have wished (39.9%); however, more than one-quarter of sites which responded were found to have experience of administering bevacizumab 10 mg/kg Q2W. Moreover, to participate in this study, sites were required to obtain ethical approval, and sites which did not use this regimen may have been less likely to apply for ethical approval and respond to the questionnaire. Third, as we asked physicians only about the efficacy and safety of bevacizumab 10 mg/kg administered Q2W, and we did not collect efficacy/safety data relating to the dose of 15 mg/kg administered Q3W, we are unable to make any direct comparisons between these regimens. In addition, we did not request the full clinical details for each patient, so although we were able to assess response rates, we are unable to speculate on survival durations associated with the bevacizumab 10 mg/kg Q2W treatment regimen. Similarly, although a Grade 5 AE was reported and its causal relationship to bevacizumab treatment could not be ruled out, no additional details were available for a fuller evaluation.

In conclusion, this study found that the use of a non-approved dose of bevacizumab (10 mg/kg Q2W) for platinum-resistant recurrent ovarian cancer in real-world clinical practice in Japan provided an antitumor effect and had a safety profile similar to those previously reported for this dose in studies conducted outside Japan. These findings support the use of bevacizumab 10 mg/kg Q2W for the treatment of recurrent ovarian cancer, and we believe that implementing this regimen may have beneficial effects on patients’ lives.
