# Correction to: The efficacy and safety profile of 2-weekly dosing of bevacizumab-containing chemotherapy for platinum-resistant recurrent ovarian cancer

**DOI:** 10.1007/s10147-021-02018-3

**Published:** 2021-09-09

**Authors:** Masayuki Sekine, Takayuki Enomoto, Yoh Watanabe, Hidetaka Katabuchi, Nobuo Yaegashi, Daisuke Aoki

**Affiliations:** 1 Department of Obstetrics and Gynecology, Niigata University Graduate School of Medical and Dental Sciences, 757 Ichibancho, Asahimachi-dori, Chuo Ward, Niigata, Niigata Japan; 2 Division of Obstetrics and Gynecology, Faculty of Medicine, Tohoku Medical and Pharmaceutical University, 4-4-1 Komatsushima, Aobaku, Sendai, Miyagi Japan; 3 Department of Obstetrics and Gynecology, Faculty of Life Sciences, Kumamoto University, 1-1-1 Honjo, Chuo-ku, Kumamoto, Kumamoto Japan; 4 Department of Obstetrics and Gynecology, Tohoku University Graduate School of Medicine, 2-1 Seiryo-machi, Aoba-ku, Sendai, Miyagi Japan; 5 Department of Obstetrics and Gynecology, Keio University School of Medicine, 35 Shinanomachi, Shinjuku-ku, Tokyo, Japan

## Correction to: International Journal of Clinical Oncology 10.1007/s10147-021-01996-8

The article The efficacy and safety profile of 2‑weekly dosing of bevacizumab‑containing chemotherapy for platinum‑resistant recurrent ovarian cancer, written by Masayuki Sekine, Takayuki Enomoto, Yoh Watanabe, Hidetaka Katabuchi, Nobuo Yaegashi and Daisuke Aoki was originally published Online First without Open Access. With the author(s)’ decision to opt for Open Choice the copyright of the article changed on August 30, 2021 to © Author(s) 2021 and the article is forthwith distributed under a Creative Commons Attribution 4.0 International License, which permits use, sharing, adaptation, distribution and reproduction in any medium or format, as long as you give appropriate credit to the original author(s) and the source, provide a link to the Creative Commons licence, and indicate if changes were made. The images or other third party material in this article are included in the article’s Creative Commons licence, unless indicated otherwise in a credit line to the material. If material is not included in the article’s Creative Commons licence and your intended use is not permitted by statutory regulation or exceeds the permitted use, you will need to obtain permission directly from the copyright holder. To view a copy of this licence, visit http://creativecommons.org/licenses/by/4.0.

The original article has been corrected.

